# A Sole Case of Concurrent Arterial and Venous Thromboses with Massive Pulmonary Embolism and Carriage of Four Genetic Polymorphisms: Factor V Leiden, PAI-1 4G/5G, MTHFR C677T, and ACE I/D—A Case Report

**DOI:** 10.3390/reports8030167

**Published:** 2025-09-01

**Authors:** Nevena Ivanova

**Affiliations:** 1 Department of Urology and General Medicine, Faculty of Medicine, Medical University of Plovdiv, 4000 Plovdiv, Bulgaria; nevenai@yahoo.com; Tel.: +35-98-8913-0416; 2 St Karidad MHAT, Karidad Medical Health Center, Cardiology, 4004 Plovdiv, Bulgaria; 3 Department of Internal Diseases, Social Medicine, Physiotherapy and Rehabilitation, Disaster Medicine, Faculty of Medicine, University “Prof. Dr. Asen Zlatarov”, 8010 Burgas, Bulgaria

**Keywords:** arterial and venous thrombosis, genetic polymorphisms, Factor V Leiden, PAI-1 4G/5G, MTHFR C677T, ACE I/D DD

## Abstract

**Background and Clinical Significance:** Arterial and venous thromboses are typically distinct clinical entities, each governed by unique pathophysiological mechanisms. The concurrent manifestation of both, particularly in the setting of massive pulmonary embolism (PE), is exceptionally rare and poses significant diagnostic and therapeutic challenges. **Case Presentation:** This report describes a 61-year-old male with well-controlled hypertension and type 2 diabetes who developed extensive thromboses involving deep vein thrombosis (DVT) of the right popliteal vein, arterial thrombosis of the left iliac artery, and massive PE. The patient was initially managed conservatively, in accordance with the European Society of Cardiology (ESC) 2019 Guidelines for Acute PE, using unfractionated heparin (UFH), low-molecular-weight heparin, a direct oral anticoagulant (DOAC), and adjunctive therapy. This approach was chosen due to the absence of hemodynamic instability. However, given failed percutaneous revascularization and persistent arterial occlusion, surgical thromboendarterectomy (TEA) was ultimately required. Post hoc genetic testing was prompted by the complex presentation in the absence of classical provoking factors—such as trauma, surgery, malignancy, or antiphospholipid syndrome—consistent with recommendations for selective thrombophilia testing in atypical or severe cases. The analysis revealed four thrombophilia-associated polymorphisms: heterozygous Factor V Leiden (FVL; R506Q genotype), Plasminogen Activator Inhibitor-1 (PAI-1; 4G/5G genotype), Methylenetetrahydrofolate reductase (MTHFR; c.677C > T genotype), and homozygous Angiotensin-Converting Enzyme Insertion/Deletion (ACE I/D; DD genotype). **Conclusions:** While each variant has been individually associated with thrombotic risk, their co-occurrence in a single patient with simultaneous arterial and venous thromboses has not, to our knowledge, been previously documented. This case underscores the potential for gene–gene interactions to amplify thrombotic risk, even in the presence of variants traditionally considered to confer only modest to moderate risk. It highlights the need for a multidisciplinary approach and raises questions regarding pharmacogenetics, anticoagulation, and future research into cumulative genetic risk in complex thrombotic phenotypes.

## 1. Introduction and Clinical Significance

The simultaneous occurrence of arterial and venous thrombosis, particularly when complicated by massive pulmonary embolism, represents an exceedingly rare and clinically significant phenomenon, scarcely described in the medical literature [1,2,3]. Traditionally, arterial and venous thrombotic events are considered distinct entities—arterial thrombosis arising from atherosclerotic plaque rupture and platelet aggregation, and venous thrombosis resulting from hypercoagulability, stasis, and endothelial dysfunction (Virchow’s triad) [4,5]. However, growing evidence suggests that this dichotomy may converge in certain high-risk individuals, particularly those with underlying genetic predispositions [6,7].

Among inherited thrombophilias, Factor V Leiden (R506Q) and the prothrombin G20210A mutation represent the most widely validated genetic determinants of venous thromboembolism and have also been implicated, though less consistently, in arterial events [8,9]. Other genetic variations exert subtler but mechanistically relevant effects. For example, Factor VII polymorphisms influence tissue-factor–dependent coagulation activity, thereby modulating the initiation of clotting [10,11,12]. The MTHFR C677T variant predisposes to hyperhomocysteinemia, which promotes endothelial injury and a prothrombotic state [13]. Similarly, the PAI-1 4G/5G polymorphism alters the balance between fibrinolysis and fibrin persistence through regulation of plasminogen activator inhibitor-1 levels [14], while the ACE I/D polymorphism affects vascular tone and endothelial function via renin–angiotensin system activity [15,16,17]. In addition to these common variants, rare but clinically important deficiencies of natural anticoagulants—including antithrombin (SERPINC1), protein C (PROC), and protein S (PROS1)—are well-established causes of hereditary thrombophilia, conferring a markedly elevated risk of venous thromboembolism [18,19,20,21]. These high-penetrance disorders, although less prevalent, illustrate the spectrum of inherited predispositions that can disrupt hemostatic balance.

While each of these variants alone is often associated with variable or context-dependent thrombotic risk, their coexistence illustrates the diverse mechanisms—hypercoagulability, impaired fibrinolysis, endothelial dysfunction, and altered vascular tone—through which inherited predispositions may converge to shape a markedly prothrombotic milieu. To date, however, the concurrent presence of four such genetic variants—Factor V Leiden (R506Q), MTHFR C677T, PAI-1 4G/5G, and ACE I/D (DD)—has not been reported in a patient with simultaneous arterial and venous thrombosis complicated by massive pulmonary embolism. Moreover, the interplay among these variants remains poorly understood in the context of thrombotic disease.

To our knowledge, this report presents the first documented case of such a unique combination of variants. Beyond its clinical significance, this case raises an important scientific question: can polygenic interactions, rather than isolated variants, more accurately account for extreme thrombotic phenotypes? The findings challenge conventional single-gene models of thrombophilia and support a broader, integrative perspective. This case report aims to advance the understanding of polygenic contributions to thrombosis by documenting an unprecedented genetic constellation in a real-world clinical scenario, while underscoring the potential value of personalized genetic assessment in highly atypical thrombotic presentations.

## 2. Case Presentation

A 61-year-old man with a medical history of arterial hypertension and type 2 diabetes mellitus—both well controlled with ongoing pharmacological therapy and regular clinical monitoring—presented to his general practitioner by the end of August 2024. He reported lower back pain radiating to the left inguinal region and leg, which was numb, accompanied by mild swelling of the right calf, fatigue, and palpitations persisting for approximately 10 days. The patient was immediately referred for hospitalization in a neurology department, where he received intravenous nonsteroidal anti-inflammatory therapy over several days. In early September 2024, he presented to our hospital on his own initiative, without documentation or prior records from the previous evaluation. He requested additional assessment because there had been no improvement, the pain and edema had worsened, and dyspnea had developed even with minimal physical activity in the room. Upon examination in the emergency department, the patient was suspected to have deep vein thrombosis in the right leg and pulmonary embolism and was subsequently admitted to the intensive care unit of the cardiology department. A comprehensive personal and family history revealed no prior thrombotic events, evidence of malignancy, recent trauma or surgery, prolonged immobilization, or use of hormone therapy. Apart from long-standing, well-controlled arterial hypertension and type 2 diabetes mellitus, no other chronic conditions were identified. On admission, the patient was in a compromised general condition with tachypnea (28 breaths per minute; normal range for adults: 12–20 breaths per minute), though he was alert, oriented, and communicative. On physical examination, no pathological auscultatory findings were observed in the respiratory system. The cardiovascular assessment revealed a rhythmic tachycardic heart rate of 105 beats per minute, a systolic murmur consistent with tricuspid regurgitation, and stable hemodynamics with blood pressure readings of 139/91 mmHg. Local angiological examination identified mild swelling of the right calf, a weak-to-absent pulse in the left femoral artery, and no palpable distal pulsations, with the left calf and foot appearing cool and pale.

**Figure 1:**
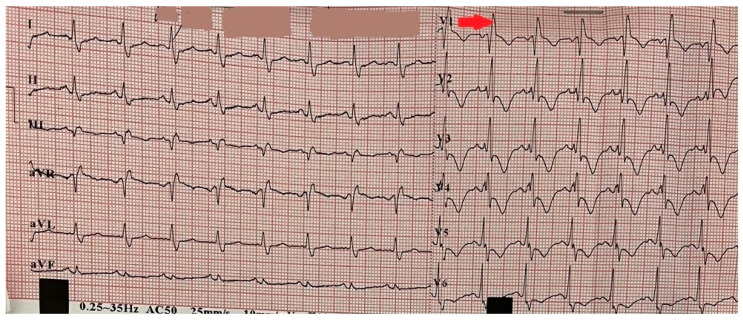
Electrocardiogram on admission demonstrating sinus tachycardia and right bundle branch block (red arrow).

Initial instrumental investigations included an electrocardiogram (ECG), which, on admission, demonstrated sinus tachycardia and right bundle branch block (Figure 1).

Laboratory evaluation demonstrated leukocytosis (13.13 × 10^9^/L; reference range: 3.5–10.5 × 10^9^/L) and significant hyperglycemia (18.17 mmol/L; reference range: 2.8–6.2 mmol/L), suggestive of systemic stress. These findings were accompanied by markedly elevated D-dimer levels (10.5 mg/L; normal < 0.5 mg/L) and a substantial increase in troponin I (6.64 ng/mL; reference range: 0.0–0.1 ng/mL), collectively indicative of extensive thrombotic activity and myocardial strain, consistent with acute pulmonary embolism and right ventricular dysfunction. A comprehensive panel of routine coagulation tests was conducted, encompassing Activated Partial Thromboplastin Time (aPTT), International Normalized Ratio (INR), fibrinogen levels, and platelet count, with results falling within the respective reference ranges. Additionally, a standard lipid profile, including total cholesterol, low-density lipoprotein (LDL), high-density lipoprotein (HDL), and triglycerides, revealed values consistent with normal physiological limits. Oxygen saturation was 88% (normal range: 94–100%), and arterial blood gas analysis indicated hypoxemia with reduced partial oxygen pressure of 65 mmHg (normal range: 75–100 mmHg), hypocapnia of 28 mmHg (normal range: 35–45 mmHg), and respiratory alkalosis with a pH of 7.49 (normal range: 7.35–7.45).

**Figure 2:**
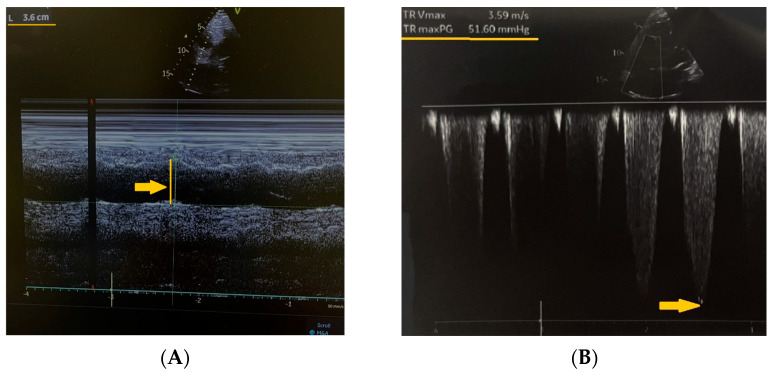
(**A**,**B**) Echocardiography revealing a dilated right ventricle 36 mm in the parasternal long-axis view and a maximal tricuspid valve gradient of 51.6 mmHg measured by continuous wave Doppler (yellow arrows).

Further diagnostic workup included echocardiography, which revealed a dilated right ventricle measured at 36 mm in the parasternal long-axis view (normal range: 20–30 mm) (Figure 2A), moderate-to-severe tricuspid regurgitation, and a maximal tricuspid valve gradient of 51.6 mmHg measured by continuous-wave Doppler (normal: <10 mmHg) (Figure 2B).

**Figure 3:**
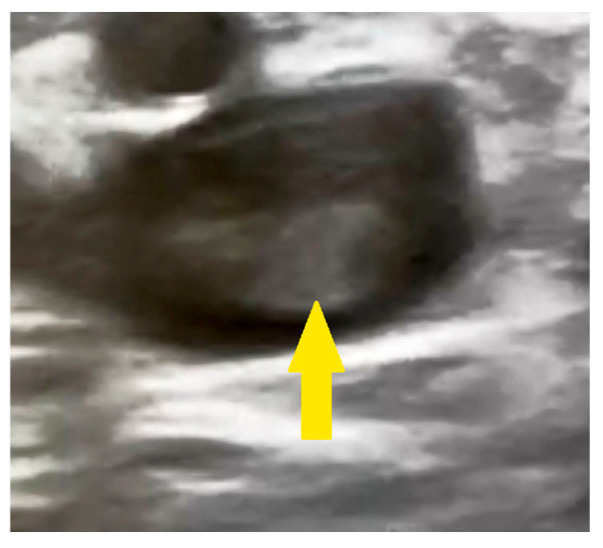
Ultrasound examination of the right calf revealing a thrombus in the popliteal vein (yellow arrow).

The estimated systolic pulmonary artery pressure was approximately 70 mmHg (normal resting value: <35 mmHg). Ultrasound examination of the right calf revealed a thrombus in the popliteal vein as documented in the surgical consultation records (Figure 3).

**Figure 4:**
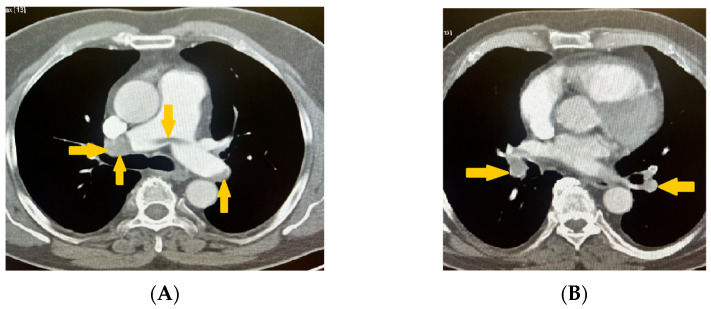
(**A**,**B**) CT pulmonary angiography with 3D reconstruction imaging the right and left pulmonary arteries exhibiting an extensive saddle-shaped filling defect with intraluminal occlusions bilaterally, including the interlobar and segmental branches (yellow arrows).

These findings supported deep vein thrombosis complicated by pulmonary embolism. The diagnosis was confirmed using non-contrast computed tomography (CT) and CT pulmonary angiography with reconstruction. Imaging studies revealed that the pulmonary trunk had homogenous contrast enhancement, which was at the upper limit of normal dimensions (<29 mm). The right and left pulmonary arteries exhibited an extensive saddle-shaped filling defect (Figure 4A) with intraluminal occlusions bilaterally, including the interlobar and segmental branches (Figure 4B).

**Figure 5:**
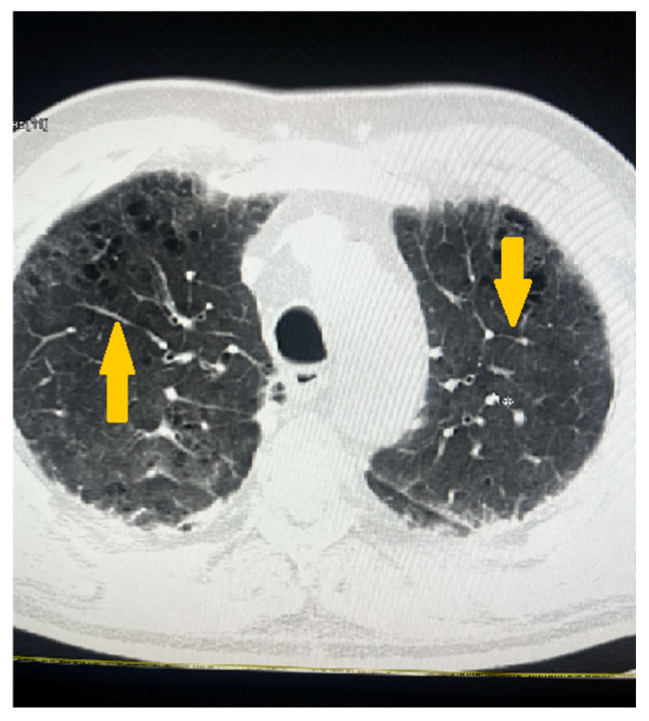
Non-contrast computed tomography (CT) showing the pulmonary parenchyma with prominent broncho-vascular markings (yellow arrows).

The pulmonary parenchyma showed prominent broncho-vascular markings without evidence of infiltrative or focal lesions (Figure 5).

**Figure 6:**
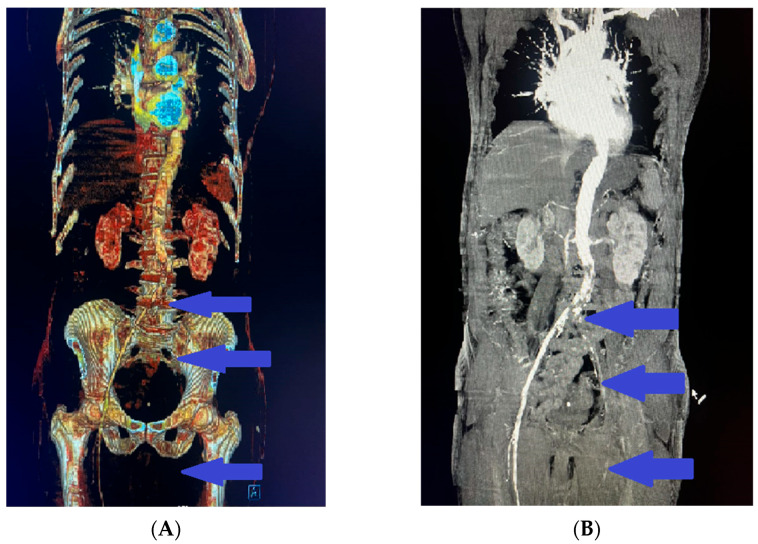
(**A**,**B**) Contrast computed tomography (CT) angiography scans revealing complete obliteration and no contrast enhancement in the left iliac artery (blue arrows).

Based on the patient’s history of pain and numbness in the left leg, combined with the objective pathological findings, additional imaging of the caudal sections was analyzed. The scans revealed complete obliteration and no contrast enhancement in the left iliac artery (Figure 6A,B). Clinically, this presentation was consistent with Rutherford stage IIa–IIb acute limb ischemia (sensory deficit without major motor involvement), although this classification was not explicitly recorded in the surgical notes.

Given the simultaneous involvement of both the venous and arterial systems, including massive pulmonary embolism, a decision regarding fibrinolytic therapy was made in accordance with the 2019 European Society of Cardiology Guidelines for the Diagnosis and Management of Acute Pulmonary Embolism. This document recommends systemic fibrinolysis only in hemodynamically unstable patients (Class I, Level A), unless contraindications exist [22]. In this case, the patient was stratified as intermediate–high risk, with a total of 5 points according to advanced risk assessment scores: right ventricular dysfunction on imaging (2 points), elevated cardiac troponin levels (2 points), and a heart rate > 110 bpm (1 point) [22]. However, he remained hemodynamically stable, with no signs of shock or systolic blood pressure < 90 mmHg, thus not fulfilling the criteria for high-risk pulmonary embolism requiring fibrinolysis. This decision was further supported by the findings of the PEITHO trial, which demonstrated that, although thrombolysis reduced the incidence of hemodynamic decompensation in normotensive patients with intermediate-risk PE, it also significantly increased the risk of major and intracranial bleeding [23]. Taken together, these considerations reinforced the choice to manage the patient with anticoagulation. Treatment was initiated with unfractionated heparin (UFH), administered as a 10,000 unit intravenous (i.v.) bolus, followed by a continuous infusion at 1000 units per hour, with activated partial thromboplastin time (aPTT) closely monitored and maintained within the therapeutic range of 46–70 s (normal range: 25–35 s). Platelet levels were also tracked to monitor for potential heparin-induced thrombocytopenia (HIT). This initial anticoagulation approach aligned with the 2019 European Society of Cardiology Guidelines, which recommend parenteral anticoagulation as the first-line treatment in intermediate-high-risk pulmonary embolism when thrombolysis is not indicated due to hemodynamic stability (Class IIa, Level B) [22].

After 48 h of UFH, therapy was transitioned to fondaparinux (10 mg subcutaneously once daily), a synthetic factor Xa inhibitor with a more predictable anticoagulant response, lower risk of HIT, and no need for laboratory monitoring [22]. This change was made to enhance patient safety and treatment efficacy.

Supplemental oxygen was delivered via a face mask at a rate of 10 L per minute, and ceftriaxone sodium was administered at a dose of 1 g i.v. twice daily.

**Figure 7:**
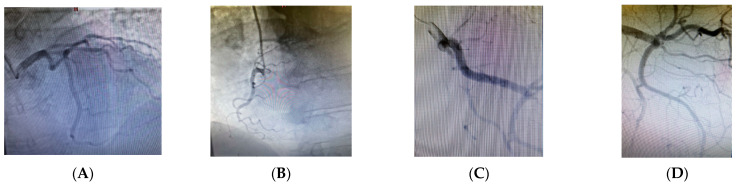
(**A**–**D**) Angiographic imaging of the coronary arteries showing no evidence of atherosclerotic plaques.

Due to the underlying DVT, the right lower extremity was bandaged with an elastic compression bandage. In response to the ischemia of the left lower extremity, intravenous treatment with pentoxifylline was initiated at a dose of 200 mg diluted in 500 mL of normal saline, administered over 60 min. The decision to initiate pentoxifylline therapy was based on clinical judgment in the context of acute limb ischemia, where immediate improvement in microcirculation was critical. Although not a first-line treatment for acute arterial thrombosis, pentoxifylline has demonstrated efficacy in peripheral vascular disease through its hemorheological effects, enhancing red blood cell flexibility, reducing blood viscosity, and decreasing platelet aggregation. These mechanisms improve tissue oxygenation and perfusion in ischemic regions, justifying its use as a supportive measure in this urgent clinical scenario [24]. Given the persistence of ischemic signs in the left lower extremity and the high risk of surgical intervention, invasive angiographic evaluation was performed through access via the right radial artery. This approach was undertaken after initial stabilization and prioritization of anticoagulation therapy. Angiographic imaging of the coronary arteries showed no evidence of atherosclerotic plaques (Figure 7A–D). The decision to perform coronary angiography was driven by the need to ensure cardiovascular safety before potential surgical revascularization [25], particularly in light of the possibility that percutaneous peripheral artery revascularization might fail. In the context of acute limb ischemia and multiple cardiovascular risk factors, it was essential to exclude significant coronary artery disease before undertaking high-risk vascular surgery [26,27]. Furthermore, the decision to perform coronary angiography was justified by evidence from a study demonstrating that 55% of patients with symptomatic lower limb arterial disease also had significant, yet clinically silent, coronary artery disease, highlighting the importance of proactive cardiovascular risk assessment in this population [28].

**Figure 8:**
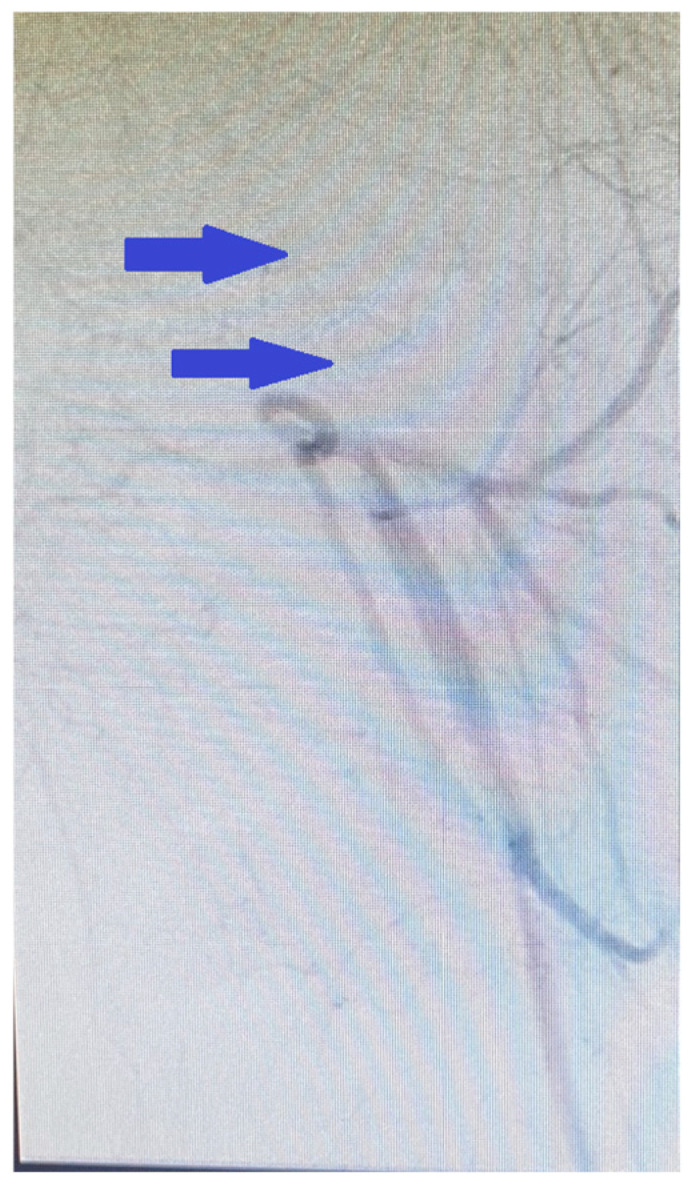
Aortography revealing occlusion of the external iliac artery, the common femoral artery, and the superficial femoral artery (blue arrows).

Subsequent aortography revealed occlusion of the external iliac artery, the common femoral artery, and the superficial femoral artery up to Hunter’s canal (Figure 8), while the arterial system of the right leg remained intact.

**Figure 9:**
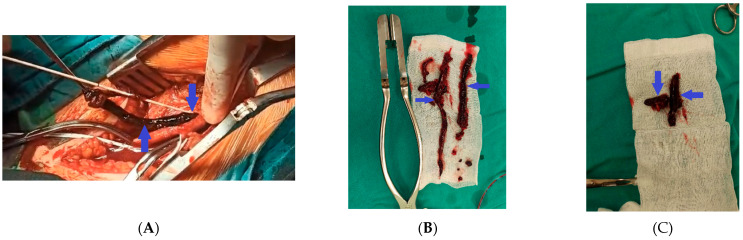
(**A**–**C**) Thromboendarterectomy (TEA) of the left iliac artery and massive thrombotic masses (blue arrows).

Attempts at percutaneous revascularization via an anterograde approach were unsuccessful, leading to termination of the procedure. Given the urgency of the clinical situation, the patient was promptly transferred to the vascular surgery department of our hospital, where operative intervention was undertaken without delay. Surgical treatment was performed under general anesthesia via an inguinal approach, including direct proximal thromboendarterectomy of the left iliac artery (8–10 cm) (Figure 9A), profunda plasty (10–12 cm), and distal TEA (50 cm). Massive thrombotic masses were evacuated (Figure 9B,C), resulting in satisfactory pulse restoration and retrograde blood flow.

Before discharge, an ultrasound examination documented Doppler signals in the pedal arteries of the left foot. The patient was advised to continue home treatment with a direct oral anticoagulant (edoxaban, 60 mg daily). Edoxaban was selected not only for its established clinical efficacy in reducing recurrent venous thromboembolism, as demonstrated in the Hokusai-VTE trial, but also for its favorable safety profile and once-daily dosing regimen [29]. Importantly, in the context of a suspected underlying prothrombotic or hereditary thrombophilic state-such as Factor V Leiden mutation-edoxaban offers a distinct advantage, as it does not reduce plasma levels of natural anticoagulants including protein C and protein S. This pharmacodynamic property renders it particularly suitable for patients in whom preservation of these endogenous inhibitors of coagulation is clinically relevant. Edoxaban demonstrated that it significantly reduces thrombotic burden while maintaining stable levels of protein C and S, further reinforcing its appropriateness in individuals with inherited thrombophilic disorders [30].

In addition, nattokinase (200 mg daily) was recommended based on its safety and potential benefit in patients with vascular diseases, particularly for improving circulation and reducing thrombotic burden without significant adverse effects [31]. Diosmin (1000 mg daily) was prescribed in light of evidence from a randomized, double-blind, placebo-controlled trial demonstrating its efficacy in improving symptoms and quality of life in patients with chronic venous disease [32].

The patient was consulted pre- and postoperatively with a cardiologist, who recommended testing for antiphospholipid syndrome (APS) and inherited thrombophilia, given the extensive and unusual involvement of both the arterial and venous systems. These tests were subsequently performed in the outpatient setting following the patient’s discharge from the hospital.

The diagnosis of antiphospholipid syndrome was excluded in accordance with the revised Sapporo (Sydney) classification criteria [33], which require the presence of both clinical and laboratory features. The full antiphospholipid antibody (aPL) panel—including lupus anticoagulant (LAC), anticardiolipin antibodies (aCL; IgG and IgM), and anti-β2 glycoprotein I antibodies (anti-β2GPI; IgG and IgM)—was negative on two separate occasions, 12 weeks apart. This approach aligns with current best practices and reflects the updated principles of the 2023 American College of Rheumatology (ACR) and European Alliance of Associations for Rheumatology (EULAR) classification framework, which emphasizes a domain-based assessment incorporating both clinical and serological criteria [34].

The genetic analysis found that the patient was a carrier of four mutations in genes related to coagulation-factor V Leiden: heterozygous carrier of R506Q (F5 gene, p. R506Q); PAI-1: heterozygous carrier, 4G/5G genotype, MTHFR gene: heterozygous carrier, C/T genotype (c.677C>T); ACE I/D: homozygous carrier, D/D genotype. Testing for the Prothrombin (F2) gene (c.20210G>A) demonstrated a normal genotype. Additional outpatient laboratory evaluation revealed elevated blood homocysteine levels of 29 µmol/L (normal range: 5–15 µmol/L; moderately elevated: 15–30 µmol/L), consistent with heterozygous carriage of the MTHFR polymorphism.

Assessment for protein C and protein S deficiencies was not performed during hospitalization, as the patient was treated with unfractionated heparin and subsequently low molecular weight heparin, both of which significantly influence the accuracy of functional assays for natural anticoagulants. Although direct oral anticoagulants, such as edoxaban, exert a lesser impact on these assays compared to vitamin K antagonists, they can still interfere with test interpretation. Current guidelines recommend deferring such evaluations until at least three months after the thrombotic event, when anticoagulation can be safely discontinued, as early testing may yield unreliable results, necessitate repetition, and does not alter immediate management [35,36]. Given the patient’s high thrombotic risk, interruption of anticoagulation was considered clinically inappropriate.

## 3. Discussion

To establish the novelty of this presentation, a comprehensive literature search was conducted across PubMed, Scopus, and EMBASE from their inception to 28 June 2025. Targeted Boolean strategies were applied, combining terms such as “simultaneous arterial and venous thrombosis” OR “concurrent arterial and venous thrombosis”; “arterial thrombosis” AND “venous thrombosis” AND “massive pulmonary embolism”; and genetic keywords including “thrombophilia,” “thrombophilic polymorphisms,” “Factor V Leiden,” “MTHFR C677T,” “PAI-1 4G/5G,” and “ACE I/D.” Additional terms, such as “hyperhomocysteinemia,” “multiple genetic thrombophilia,” and “multiple genetic mutations and thrombosis,” were also explored. Despite this extensive search strategy, no previously published case was identified that described the precise constellation of genetic findings observed in the present report, confirming its uniqueness within the literature.

Simultaneous arterial and venous thrombosis is an uncommon clinical presentation that has been documented in various case reports [37,38,39,40]. Despite the differences in the mechanisms underlying thrombotic complications in the arterial and venous systems, these events result from the interplay of numerous environmental factors (such as surgical interventions, smoking, hormonal disorders, diabetes mellitus, arterial hypertension, dyslipidemia, malignancies, and others) with hereditary (genetic) predispositions. This interaction ultimately leads to coagulation disturbances [41].

This case represents a rare and compelling example of how multiple thrombophilia-associated genetic polymorphisms, in conjunction with acquired cardiovascular risk factors, likely converge to produce an exceptionally severe and clinically unique thrombotic phenotype involving both the arterial and venous systems. The simultaneous occurrence of arterial and venous thrombosis remains an extremely rare phenomenon [1,2,3]. The uniqueness of this case is amplified by the post hoc identification of four thrombophilia-associated genetic polymorphisms—Factor V Leiden, PAI-1 4G/5G, MTHFR C677T, and ACE I/D DD—which together may have exerted a synergistic prothrombotic influence, potentially contributing to the development of life-threatening thrombotic events, including massive pulmonary embolism and peripheral arterial occlusion.

While each of these polymorphisms has been individually studied in the context of thrombosis, their concurrent presence in a single patient with chronic cardiovascular comorbidities such as hypertension and type 2 diabetes—but without major transient or classical provoking factors such as malignancy, surgery, trauma, antiphospholipid syndrome or prolonged immobilization—raises important clinical questions. Specifically, it prompts consideration of their combined pathophysiological significance, potential gene–gene interactions, and the magnitude of cumulative thrombotic risk.

Factor V Leiden represents the most frequently identified hereditary thrombophilia in Caucasian populations, with a prevalence estimated at 1% to 5% [42]. It is characterized by a point mutation in the F5 gene, resulting in the substitution of guanine with adenine at position 1691, which leads to an amino acid change from arginine to glutamine at position 506 (p.Arg506Gln). This genetic alteration renders coagulation factor V resistant to inactivation by activated protein C (APC), thereby impairing an essential physiological anticoagulant pathway and resulting in increased thrombin generation and a hypercoagulable state [43]. Clinical data have shown that this mutation is present in approximately 10% to 20% of patients with venous thromboembolism [44], conferring a 7-fold increased risk of deep vein thrombosis and a 3-fold increased risk of pulmonary embolism [44]. Although traditionally associated with venous thrombotic events, growing evidence implicates Factor V Leiden in arterial thrombosis, particularly when additional cardiovascular risk factors are present [45,46,47]. In this case, Factor V Leiden, identified in a patient with simultaneous arterial and venous thrombosis, raises the possibility of a broader prothrombotic influence extending beyond the venous system, particularly when considered as part of a polygenic risk profile. In addition to the potential impact of impaired protein C regulation due to Factor V Leiden, reduced fibrinolytic capacity associated with overexpression of Plasminogen Activator Inhibitor-1 may have further contributed to clot persistence in this patient. PAI-1 is a major regulator of fibrinolysis, acting as a serine protease that inhibits both tissue plasminogen activator (tPA) and urokinase plasminogen activator (uPA), and thereby controls the breakdown of fibrin clots [48]. The 4G/5G polymorphism in the promoter region of the PAI-1 gene affects transcriptional activity, with the 4G allele associated with increased gene expression and elevated plasma PAI-1 levels [49]. Elevated PAI-1 levels contribute to impaired fibrinolysis and likely predispose individuals to thrombotic complications [50]. Meta-analyses have demonstrated a statistically significant correlation between the PAI-1 4G/5G polymorphism and increased risk of venous thromboembolism [50,51]. Importantly, one study explicitly reported that the 4G/5G polymorphism was significantly associated with VTE risk in patients carrying the Factor V Leiden mutation [50], suggesting a synergistic gene–gene interaction that may amplify prothrombotic risk beyond the contribution of either variant alone. In the context of this case, the coexistence of both Factor V Leiden and PAI-1 4G/5G polymorphisms suggests a genetically driven predisposition to elevated PAI-1 expression, which likely impaired fibrinolytic activity and resistance to activated protein C, thereby contributing to the development of extensive arterial and venous thrombosis. Moreover, the 4G allele has been associated with ischemic stroke and a family history of coronary artery disease [52,53]. Given these established links, its presence in our patient, alongside other thrombophilic polymorphisms, likely acted synergistically to amplify thrombotic risk across both arterial and venous systems, thereby contributing to the pathogenesis of the observed thrombotic events.

The MTHFR C677T polymorphism further complicates the prothrombotic profile in this case. Although MTHFR variants are not universally accepted as independent thrombophilia markers, a substantial body of literature supports their contributory role in thrombotic pathophysiology via alterations in homocysteine metabolism. The substitution of cytosine (C) with thymine (T) at nucleotide position 677 results in an alanine-to-valine substitution at codon 222 (p.Ala222Val) in the methylenetetrahydrofolate reductase enzyme. This leads to the formation of a thermolabile isoform with reduced enzymatic activity, which impairs the remethylation of homocysteine to methionine and leads to elevated plasma homocysteine levels and decreased folate availability [54,55,56].

Hyperhomocysteinemia is considered to contribute to the pathogenesis of both arterial and venous thrombosis through several interrelated mechanisms. Elevated homocysteine levels have been shown to cause degeneration of aortic smooth muscle cells and endothelial damage [57,58,59], and to impair endothelial function by decreasing nitric oxide (NO) bioavailability, primarily through oxidative stress and the generation of reactive oxygen species (ROS), which neutralize NO and compromise endothelium-dependent vasodilation [60,61,62]. This results in vascular stiffness, leukocyte adhesion, platelet activation, and a proinflammatory milieu that promotes both thrombogenesis and atherosclerosis [63]. Homocysteine also induces the generation of superoxide and peroxynitrite, further exacerbating oxidative endothelial damage [64,65]. Moreover, hyperhomocysteinemia has been implicated in downregulation of thrombomodulin expression, an essential endothelial anticoagulant protein that activates protein C, thereby tipping the hemostatic balance toward thrombosis [66,67,68].

In experimental models, hyperhomocysteinemia has been associated with increased expression of vascular cell adhesion molecule-1 (VCAM-1), promoting leukocyte adhesion and perpetuating vascular inflammation and plaque progression [69]. Additionally, disturbances in lipid metabolism associated with hyperhomocysteinemia, such as hepatic steatosis and accumulation of cholesterol esters and triglycerides in vascular tissues, may further accelerate atherogenesis [70].

From a clinical standpoint, multiple studies have described associations between MTHFR polymorphisms and thrombotic events. Case reports have documented pulmonary embolism [71,72,73] and peripheral arterial involvement [74] in patients with MTHFR mutations. A retrospective chart review demonstrated that both the C677T and A1298C polymorphisms were associated with increased risk of venous thromboembolism (VTE) [75]. Furthermore, meta-analyses have identified hyperhomocysteinemia as an independent risk factor for VTE [76], and some studies suggest that even moderately elevated homocysteine levels may increase thrombotic risk [77]. A large-scale meta-analysis of 37 studies indicated that the MTHFR C677T polymorphism may increase VTE risk, particularly in certain populations, such as Asians, highlighting the possibility of ethnic variability in gene expression and interaction [78]. Notably, a 2023 case series reported thrombotic events in neonates with MTHFR variants, raising concern for a broader spectrum of clinical impact in critically ill populations. The authors observed that all affected patients in their unit carried MTHFR mutations, highlighting a potential association between these variants and heightened thrombotic risk. They proposed that MTHFR mutations should be considered a risk factor in critically ill patients and advocated for routine screening in all intensive care unit admissions to aid in early risk identification [79]. In this patient, homocysteine was moderately elevated at 29 µmol/L, a concentration that has been reported to be potentially associated with increased thrombotic risk. The presence of the MTHFR C677T polymorphism, in conjunction with elevated homocysteine, likely contributed to endothelial dysfunction and a systemic prothrombotic state. Moreover, homocysteine has been shown to upregulate PAI-1 expression via Nuclear Factor kappaB (NF-κB)-mediated inflammatory signaling [80], further impairing fibrinolysis. When considered alongside other thrombophilic polymorphisms—PAI-1 4G/5G, Factor V Leiden, and ACE I/D—this creates a synergistic pathophysiological profile in which coagulation activation, endothelial injury, and reduced fibrinolytic capacity likely collectively contributed to the widespread thrombosis observed in this patient.

In continuation of the prothrombotic cascade likely promoted by hyperhomocysteinemia and endothelial dysfunction related to the MTHFR C677T variant, the ACE I/D polymorphism may have added a further mechanistic layer to the patient’s vascular vulnerability. This polymorphism, located in intron 16 of the ACE gene, involves the insertion (I) or deletion (D) of a 287 base pair fragment. Homozygosity for the D allele (DD genotype) is associated with approximately 40–50% elevated circulating levels of angiotensin-converting enzyme (ACE), which in turn promotes the production of angiotensin II, a key effector peptide in the renin–angiotensin system [81,82,83]. Angiotensin II exerts pleiotropic effects, including vasoconstriction, increased oxidative stress, proinflammatory cytokine release, endothelial dysfunction, and vascular smooth muscle cell proliferation, which processes underlie the progression of cardiovascular diseases such as atherosclerosis and hypertension [17,84,85]. Some researchers suggest that this genotype is less related to the formation of stenosis and more to thrombogenesis mechanisms and the imbalance between thrombus formation and fibrinolysis [86,87]. Furthermore, experimental studies have shown that angiotensin II increases PAI-1 messenger ribonucleic acid (mRNA) levels and plasma PAI-1 concentrations [88,89,90], as well as PAI-1 expression in vascular endothelial and smooth muscle cells [91]. This impaired endogenous fibrinolysis, mediated by PAI-1, has been linked to an elevated risk of intravascular thrombosis [92]. In the presented patient, the presence of the ACE I/D DD genotype likely exacerbated the effects of the PAI-1 4G/5G polymorphism and hyperhomocysteinemia by promoting a proinflammatory, prothrombotic, and antifibrinolytic vascular environment. The convergence of these mechanisms may have critically contributed to the pathogenesis of both venous and arterial thrombosis. Regarding the effects of the ACE I/D polymorphism on coagulation, numerous studies have investigated its association with venous thromboembolism [93,94,95,96]. While a meta-analysis involving approximately 7000 participants did not confirm a direct association between the ACE I/D polymorphism and VTE, it highlighted the possibility of gene–gene and gene–environment interactions that could amplify thrombotic risk in genetically predisposed individuals [97]. This supports the hypothesis that the DD genotype, though of modest risk in isolation, may act synergistically with other variants to promote clinically significant thrombotic events, as observed in this case.

The cumulative impact of the identified polymorphisms—Factor V Leiden, PAI-1 4G/5G, MTHFR C677T, and ACE I/D DD—appears to have resulted in a unique and clinically severe thrombotic phenotype in this patient. Although each variant is individually associated with modest to moderate risk, their combined effect likely disrupted vascular homeostasis through converging mechanisms involving impaired anticoagulant regulation, diminished fibrinolysis, and endothelial dysfunction. Taken together, these gene variants likely created a biological context favoring extensive thrombus formation across both the arterial and venous systems.

Factor V Leiden, which confers resistance to activated protein C, is considered a potential contributor to enhanced thrombin generation and a sustained procoagulant state. This procoagulant milieu could have been further influenced by the PAI-1 4G/5G polymorphism, which is associated with elevated PAI-1 levels and impaired fibrin degradation, potentially favoring clot persistence. The MTHFR C677T variant, through its link to elevated plasma homocysteine levels, may have added to vascular vulnerability by promoting oxidative stress, reducing nitric oxide bioavailability, and impairing endothelial function. Additionally, homocysteine has been shown to upregulate PAI-1 expression via NF-κB activation, which may have further intensified antifibrinolytic effects in this patient.

Adding to this, the ACE I/D polymorphism, particularly the DD genotype, likely intensified angiotensin II-mediated vasoconstriction, oxidative injury, and inflammation. Angiotensin II has also been implicated in upregulating PAI-1 expression, promoting vascular smooth muscle proliferation, and further contributing to endothelial dysfunction and thrombogenesis. Notably, angiotensin II and homocysteine share converging downstream pathways—namely, the enhancement of oxidative stress and suppression of endothelial resilience—which likely synergistically exacerbated vascular injury.

In this patient, the convergence of these mechanisms appears to underlie the simultaneous presentation of deep vein thrombosis, arterial thrombosis of the iliac and femoral arteries, and massive pulmonary embolism—an exceedingly rare and life-threatening combination. The absence of classic provoking factors, such as surgery, trauma, immobilization, malignancy, or antiphospholipid syndrome, underscores the likely pathogenic role of the observed genetic constellation, particularly when considered alongside chronic cardiovascular comorbidities such as hypertension and type 2 diabetes mellitus. The moderate elevation of homocysteine (29 µmol/L), the coexistence of the PAI-1 4G/5G and ACE DD genotypes, and the presence of Factor V Leiden together likely collectively amplified thrombotic susceptibility through an intricate interplay of endothelial dysfunction, impaired fibrinolysis, and sustained hypercoagulability.

In accordance with the British Society for Haematology guidelines, thrombophilia testing is not routinely recommended for all patients but may be considered in individuals with unprovoked, recurrent, or unusual-site thrombosis, especially when the clinical presentation is atypical or severe [27]. In this patient, the simultaneous occurrence of arterial and venous thrombosis, massive pulmonary embolism, and the absence of common provoking factors raised legitimate concern for an underlying inherited prothrombotic predisposition. Thus, a targeted genetic evaluation was performed post hoc to investigate potential contributing factors, consistent with best-practice guidance and not as part of routine screening. While existing guidelines provide a robust framework for thrombophilia testing, they also allow for clinician discretion in exceptional cases. In the context of this rare and clinically complex presentation, genetic evaluation was undertaken judiciously, reflecting the need for individualized decision-making in situations where conventional paradigms may be insufficient to fully elucidate the underlying pathophysiological mechanisms. Although familial genetic testing was not undertaken in this case, the identification of a complex polygenic predisposition underscores a potential rationale for considering family screening in selected situations. While not recommended as routine practice by current guidelines, such an approach may, in exceptional and severe phenotypes, facilitate early risk detection and preventive strategies in relatives.

This case provides mechanistic insight into how multiple thrombophilia-associated polymorphisms appear to interact to produce a markedly prothrombotic phenotype. It illustrates that pathogenicity may not arise from a single variant but from the cumulative biological effects of gene–gene and gene–environment interactions. Understanding such integrative pathophysiology is essential for deciphering atypical thrombotic presentations and refining future genomic risk models. However, the long-term clinical implications could not be determined, as no follow-up data were available. The patient did not attend scheduled outpatient visits and could not be contacted after discharge. This limitation, together with the lack of published data on similar polygenic thrombotic profiles, precludes assessment of long-term outcomes but underscores the need for systematic follow-up in future reports.

## 4. Conclusions

This case presents, to our knowledge, the first documented instance of simultaneous arterial and venous thrombosis in a patient carrying a unique constellation of four thrombophilia-associated genetic polymorphisms. It underscores how the coexistence of multiple variants—each typically conferring only modest thrombotic risk—may interact synergistically, with cumulative effects exceeding those of any single mutation and amplifying endothelial dysfunction, hypercoagulability, and fibrinolytic resistance. These observations highlight the need for prospective studies to clarify polygenic risk, gene–gene interactions, and their implications for personalized anticoagulation and long-term management strategies in complex thrombotic disorders.

## Data Availability

The data presented in this study are available on request from the corresponding author. The data are not publicly available due to privacy/ethical reasons.

## Abbreviations

The following abbreviations are used in this manuscript:

PE	pulmonary embolism
DVT	deep vein thrombosis
DOACs	direct oral anticoagulants
VTE	venous thromboembolism
FVL	Factor V Leiden
MTHFR	methylenetetrahydrofolate reductase
PAI-1	plasminogen activator inhibitor-1
I/D	insertion/deletion
ACE	angiotensin-converting enzyme
ECG	electrocardiogram
CT	computed tomography
aPTT	activated partial thromboplastin time
HIT	heparin-induced thrombocytopenia
TEA	thromboendarterectomy
tPA	tissue plasminogen activator
F	factor
APS	antiphospholipid syndrome
aPL	antiphospholipid antibody
LAC	lupus anticoagulant
aCL	anticardiolipin antibodies
anti-β2GPI	anti-β2 glycoprotein I antibodies
ACR	American College of Rheumatology
EULAR	European Alliance of Associations for Rheumatology
APC	activated protein C
uPA	urokinase plasminogen activator
4G/5G	guanosine polymorphism
C	cytosine
T	thymine
NO	nitric oxide
ROS	reactive oxygen species
VCAM-1	vascular cell adhesion molecule-1
NF-κB	Nuclear Factor- kappaB
mRNA	messenger ribonucleic acid
